# Emodin Inhibits the Proliferation of MCF-7 Human Breast Cancer Cells Through Activation of Aryl Hydrocarbon Receptor (AhR)

**DOI:** 10.3389/fphar.2020.622046

**Published:** 2021-01-19

**Authors:** Ning Zhang, Jiawen Wang, Aimin Sheng, Shuo Huang, Yanyan Tang, Shitang Ma, Ge Hong

**Affiliations:** ^1^ Life and Health College, Anhui Science and Technology University, Fengyang, China; ^2^ School of Chemical Engineering, Anhui University of Science and Technology, Huainan, China; ^3^ Tianjin Key Laboratory of Biomedical Materials, Institute of Biomedical Engineering, Chinese Academy of Medical Science and Peking Union Medical College, Tianjin, China; ^4^ School of Pharmacy, East China University of Science and Technology, Shanghai, China; ^5^ Clinical College of Orthopedics, Tianjin Medical University, Tianjin Hospital, Tianjin, China

**Keywords:** emodin, AhR, CYP1A1, breast cancer, MCF-7, network pharmacology

## Abstract

Natural products have proved to be a promising source for the development of potential anticancer drugs. Emodin, a natural compound from *Rheum palmatum*, is used to treat several types of cancers, including lung, liver, and pancreatic. However, there are few reports regarding its use in the treatment of breast cancer. Thus, the therapeutic effect and mechanism of emodin on MCF-7 human breast cancer cells were investigated in this study. Morphological observations and cell viability were evaluated to determine the anti-proliferation activity of emodin. Network pharmacology and molecular docking were performed to screen the potential targets. Western blot analysis was used to explore a potential antitumor mechanism. The results showed that emodin (50–100 μmol/L) could significantly inhibit the proliferation of MCF-7 cells in a time and dose-dependent manner. Furthermore, virtual screening studies indicated that emodin was a potent aryl hydrocarbon receptor (AhR) agonist in chemotherapy for breast cancer. Finally, when MCF-7 cells were treated with emodin (100 μmol/L) for 24 h, the AhR and cytochrome P450 1A1 (CYP1A1) protein expression levels were significantly upregulated compared with the control group. Our study indicated that emodin exhibited promising antitumor activity in MCF-7 cells, likely through activation of the AhR-CYP1A1 signaling pathway. These findings lay a foundation for the application of emodin in breast cancer treatment.

## Introduction

Breast cancer is one of the most common malignant tumors that seriously threaten women’s health worldwide (Jia et al., 2015). Although screening for breast cancer is gradually being more regularly performed, incidence of the disease is still increasing year by year. According to the latest data from the International Agency for Research on Cancer (IARC) in 2018, breast cancer ranks first among female cancers worldwide with an incidence rate of 24.2%. Notably, 52.9% of these breast cancer cases occur in developing countries and seriously threaten the lives of the patients (Ferlay et al., 2019). With the increasing incidence of breast cancer in women, surgery is currently the main clinical treatment, but adjuvant chemotherapy is also widely used in breast cancer patients. These treatment strategies are relatively expensive. For example, the cost of breast cancer care in the United States in 2020 was predicted to be $19 billion (Mariotto et al., 2011). Due to its high incidence, breast cancer treatment accounts for the largest proportion of total expenditures of all cancers (Pickle et al., 2007). In addition, there is a high risk of operation failure. If not handled properly, this will endanger the life of the patient. Additionally, surgical treatment can damage the physical and mental health of female patients. Chemotherapy may cause breast cancer cells to develop multi-drug resistance (MDR), leading to therapeutic failure (Khongkow et al., 2016b). Anthracyclines, paclitaxel, and their semi-synthetic derivatives have achieved good results in the treatment of breast tumors after surgery. However, the adverse reactions of anthracyclines and taxanes, especially hematological adverse reactions (myelosuppression) and drug resistance, negatively affected effective application of these drugs. These side effects were also one of the common reasons for the failure of breast cancer chemotherapy (Khongkow et al., 2016a). Therefore, discovery of effective adjuvant therapy and alternative therapy drugs has become a key focus in postoperative treatment research for breast cancer.

Emodin is a natural anthraquinone compound with anti-tumor activity and is obtained from the separation and purification of Chinese medicine *Rheum palmatum*, *Polygonum cuspidatum*, and *Aloe vera Berg* (Yang et al., 2014a). Like the anti-tumor drug mitoxantrone and antibiotic daunorubicin, emodin has no amino sugar structure or free radicals, so it has minimal adverse reactions in patients. Emodin can also induce DNA fragmentation in cancer cells (Xing et al., 2015). It has been reported that emodin has anti-inflammatory, anti-bacterial, and anti-tumor effects. Currently, published studies have focused on the role of emodin in the following cancers: ovarian cancer (Li et al., 2009; Lu et al., 2017), lung cancer (Ko et al., 2010), chronic myeloid leukemia (Chun-Guang et al., 2010), and sensitizing gastric cancer (Cai et al., 2008). For the treatment of breast cancer, some researchers have studied the estrogen receptor alpha-mitogen-activated protein kinases/Akt-cyclin D1/B-cell lymphoma-2 signaling pathway (Sui et al., 2014), regulation of myeloid cell leukemia-1, cyclin D1, and C-myc genes (Li et al., 2013), and more (Sun et al., 2015; Zu et al., 2015). However, there are few studies on emodin-mediated regulation of the AhR target of breast cancer. The potential anti-tumor and immune regulation mechanisms of emodin have become a new research hotspot.

Our study was performed to explore the mechanism by which emodin acts on breast cancer cells through a comprehensive network pharmacology and experimental approach, which proved that emodin can activate AhR to inhibit the proliferation of MCF-7 cells and promote apoptosis. This provided a basis for the clinical application of emodin and provided a reference for further research in the future.

## Materials and Methods

**FIGURE 1:**
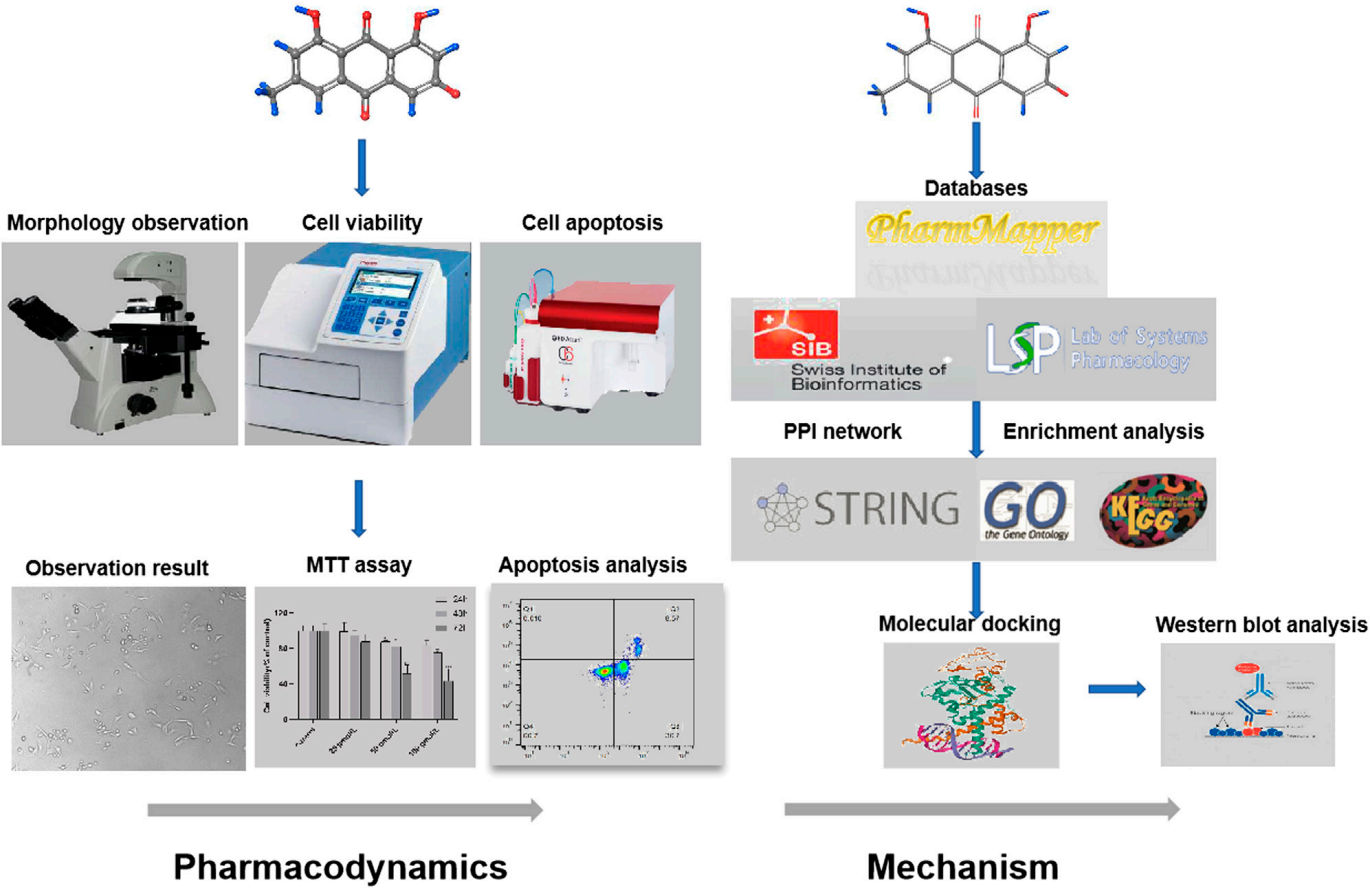
The research flowchart.

This study includes the main steps as follows:

- Chemicals and materials.
- Cell morphology observations.
- Cell proliferation was detected by MTT assay.
- Flow cytometric analysis of apoptosis.
- Chemical structure information and pharmacokinetic parameters of Emodin.
- Emodin target prediction by network pharmacology.
- Construction of Protein-Protein interaction (PPI) network.
- GO and KEGG pathways enrichment analyses.
- Molecular docking study.
- Western blot analysis. The research flowchart is shown in Figure 1.

### Chemicals and Materials

Emodin was purchased from Shanghai Yuanye Bio-Technology Co., Ltd. (Shanghai, China, HPLC≥98%, Lot: P02N6F5299), dissolved in dimethyl sulfoxide (DMSO) to prepare a stock solution of 1 mmol/L, and was stored at -20°C. MCF-7 cells were obtained from the Institute of Biomedical Engineering, Chinese Academy of Medical Sciences (Tianjin, China). For cell culture, Dulbecco’s Modified Eagle Medium (DMEM) was purchased from ThermoFisher Biochemical Products Co., Ltd. (Beijing, China). Fetal bovine serum (FBS) and phosphate-buffered saline (PBS) were purchased from Biological Industries Israel Beit Hemek Ltd (Kibbutz Beit Hamek, Israel). Streptomycin (100 μg/ml) and Penicillin (100 units/mL) were purchased from Life Technologies Corporation (New York, United States). The AhR antibody was purchased from Affinity Biosciences (Columbus, United States, Cat. No. AF6278) and the CYP1A1 antibody was purchased from Wuhan Sanying Biotechnology Co., Ltd. (Wuhan, China, Cat. No. 13241-1-AP). The secondary goat anti-rabbit antibody was purchased from Invitrogen Corporation (Carlsbad, United States). The glyceraldehyde phosphate dehydrogenase (GAPDH) antibody was obtained from Kangchen Bio-Technology Co., Ltd. (Shanghai, China).

### Cell Culture

MCF-7 cells were cultured in DMEM supplemented with 10% FBS and 1% penicillin-streptomycin. The cultures were incubated at 37°C in a humidified atmosphere containing 5% carbon dioxide. The medium was changed every two days and cells were passaged at a dilution of 1:4.

### Cell Morphology Observations

For the morphological observation of MCF-7 cells, 300,000 cells were seeded in a 6-well plate at 50,000 cells per well. Cells were treated with emodin at concentrations of 0 μmol/L, 25 μmol/L, 50 μmol/L, and 100 μmol/L. After incubating for 24 h, the cells were observed and pictures were taken using an Inverted Fluorescence Microscope (Nikon, Tokyo, Japan) in the visual field at 200×.

### MTT Assay Detect Cell Proliferation

MCF-7 cell proliferation rates were evaluated by MTT assay. Cells were plated in two 96-well plates at a density of 10^5^ cells/mL and cultured for 24 h. Cells were treated with different concentrations of emodin (0, 25, 50, and 100 μmol/L) and different concentrations of emodin (0, 25, 50, and 100 μmol/L) containing 10 μmol/L of AhR inhibitor (CH223191) (Eleftheriadis et al., 2016; Miliutina et al., 2017). After 24, 48, and 72 h of treatment, 50 µL of MTT reagent (1 mg/ml) was added, and the cells were then incubated for another 4 h. The culture medium was removed and the formazan crystals were dissolved in 150 µL of DMSO. The absorbance was measured at 490 nm by a multifunctional microplate spectrophotometer (Thermo Varioskan Flash 3,001, Waltham, MA, United States). The experiment was repeated three times and the mean value was taken. Cell viability rate = treatment group value/control group value ×100%.

### Flow Cytometric Analysis of Apoptosis

Apoptosis rates of MCF-7 cells were evaluated by an Annexin V-FITC/PI apoptosis detection kit (Beyotime Institute of Biotechnology, Shanghai, China). In general, MCF-7 cells were processed into a single cell suspension, counted and seeded in two 6-well plates, and incubated for 24 h at 37°C. The cells were then treated with different concentrations of emodin alone (0, 25, 50, 100 μmol/L) or different concentrations of emodin containing 10 μmol/L of CH223191. After 72 h of incubation, the cells were washed twice with cold PBS, digested with 0.25% trypsin (without ethylenediaminetetraacetic acid), transferred to a 15 ml centrifuge tube, and centrifuged at 1,000 rpm for 5 min, after which the supernatant was discarded. Next, 5×10^5^/ml cells were collected and gently resuspended in 195 μL of Annexin V-FITC binding buffer, followed by the addition of 5 μL of Annexin V-FITC and 10 μL of PI and then mixed evenly. After samples were covered with aluminum foil and incubated for 15 min at room temperature in the dark, they were placed in an ice bath and the cell apoptosis rates were detected using flow cytometry (CyFlow® Cube 6, Sysmex). Data are shown as pseudo color graphs and were analyzed using FlowJo Software (Tree Star Inc, Ashland, OR, Unites States).

### Chemical Structure Information and Pharmacokinetic Parameters of Emodin

Chemical structure information for emodin was obtained from the NCBI PubChem database (https://www.ncbi.nlm.nih.gov/pccompound/?term=emodin, PubChem CID: 3220). Absorption, distribution, metabolism, and excretion (ADME) as standards of pharmacokinetic parameters were obtained from the Traditional Chinese Medicine Systems Pharmacology (TCMSP) (https://tcmspw.com/molecule.php?qn=472) database to evaluate the pharmacokinetic features of emodin (Ru et al., 2014).

### Emodin Target Prediction by Network Pharmacology

To acquire potential targets, we used “emodin” as a keyword to search. All targets of the drug were screened out by the PharmMapper, Swiss Target Prediction, and TCMSP databases. The PharmMapper database (http://www.lilab-ecust.cn/pharmmapper/) is an open-access web server designed for identifying potential targets for a given molecule through the pharmacophore mapping approach (Wang et al., 2017). The Swiss Target Prediction database (http://www.swisstargetprediction.ch/) allows for estimation of the most probable macromolecular targets of a small molecule with a library of 370,000 known active sites of more than 3,000 proteins from three different species (Gfeller et al., 2014). The TCMSP database (https://tcmspw.com/molecule.php?qn=1097) is a unique pharmacology platform of Chinese herbal medicines that captures the relationships between drugs, targets, and diseases (Ru et al., 2014). Through combining the three databases, the most likely biological targets of emodin were predicted and the Universal Protein (Uniprot) database (https://www.uniprot.org/) was used to seek the UniProt Knowledgebase (UniProtKB), gene symbol, and gene ID (Bairoch et al., 2005).

### Construction of Protein-Protein Interaction (PPI) Network

PPI is the binding that occurs between protein molecules to form protein complexes by non-covalent bonds (De Las and Fontanillo, 2010; Athanasios et al., 2017). Firstly, the targets were entered into the STRING database (Szklarczyk et al., 2017) (https://string-db.org/) to perform the protein interaction analysis and visualization. The species was limited to *Homo sapiens*, a confidence level greater than 0.7 was retained, and the tsv. format of the PPI network results was downloaded. In the network, nodes represented the targets and edges represented interactions between the targets and compounds.

### GO and KEGG Pathway Enrichment Analyses

Gene Ontology (GO) analysis (http://geneontology.org/) was performed, which included biological process (BP), molecular function (MF), and more (Gaudet and Dessimoz, 2017). Kyoto Encyclopedia of Genes and Genomes (KEGG) pathway analysis (Zhang et al., 2019) (https://www.kegg.jp/) was also performed, which can identify the functional role and potential biological correlation of candidate targets. The DAVID database (https://david.ncifcrf.gov/) was used to enrich GO and KEGG pathways. After the enrichment was completed, R software was used to analyze and visualize the results.

### Molecular Docking Study

A molecular docking simulation was carried out using Glide modules of Schrödinger suite 2009 (Schrödinger LLC, New York, United States) (Friesner et al., 2006). In the absence of a crystalized structure of AhR-LBD, the structurally related PAS-B domain of HIF2α (PDB ID: 3F1O) was used for molecular docking analysis. Initially, the proteins were prepared using the “protein preparation wizard” including preprocessing, reviewing, modification, and refinement. In these steps, hydrogen atoms were added and all bound water molecules were removed from the protein (Sastry et al., 2013; Shaik et al., 2016). Subsequently, protein optimization was done utilizing a reorienting hydrogen bond network and minimizing the energy using the OPLS2005 force field (Harder et al., 2016). The structure of emodin was constructed using the Mastro workspace build panel and energetically minimized with the OPLS2005 force field using the ligand minimization tool. The receptor grid was generated around the native ligand with the center coordinates 2XY (x = 19.09, y = -30.43, z = 8.86) and the size of the grid box was set to 20 Å. Other default parameters were involved in the docking calculations and all calculations were performed using Extra Precision (XP) model (Mangiatordi et al., 2017). The Pose Viewer panel was employed to visualize the interactions between emodin and amino acid residues of the model protein, as well as between CH223191 and the amino acid residues of the model protein.

### Western Blot Analysis

A protein lysis solution was used to extract total protein from treated MCF-7 cells. Protein concentrations were detected using a BCA assay kit. Proteins (20 μg) were resolved by 10% sodium dodecyl sulfate-polyacrylamide (SDS-PAGE) gel electrophoresis, followed by transfer onto polyvinylidene difluoride (PVDF) membranes and blocking with 10% Tris-buffered saline and Tween 20 (TBST) for 1.5 h at room temperature. Membranes were sealed with 5% skim milk powder sealant at room temperature for 2 h, then washed with TBST twice. The membranes were then incubated overnight at 4°C with the appropriate primary antibody (AhR or CYP1A1) at a 1:1000 dilution. Finally, the membranes were incubated for 1 h with the goat anti-rabbit IgG secondary antibody at a 1:10,000 dilution and washed three times with TBST.

### Statistical Analysis

SPSS 19.0 statistical software was used to perform experimental data analysis. The data are expressed as the mean ± standard deviation (SD). The difference between each group was compared with one-way variance analysis and comparison of cell absorbance values was used in repeated measurement analysis of variance. *p* < 0.05 indicated that the difference was statistically significant. Differences in protein expression between the individual groups were analyzed using a *t*-test with Graphpad Prism eight software and used to make column charts.

## Results

### Morphological Observations of MCF-7 Cells

**FIGURE 2:**
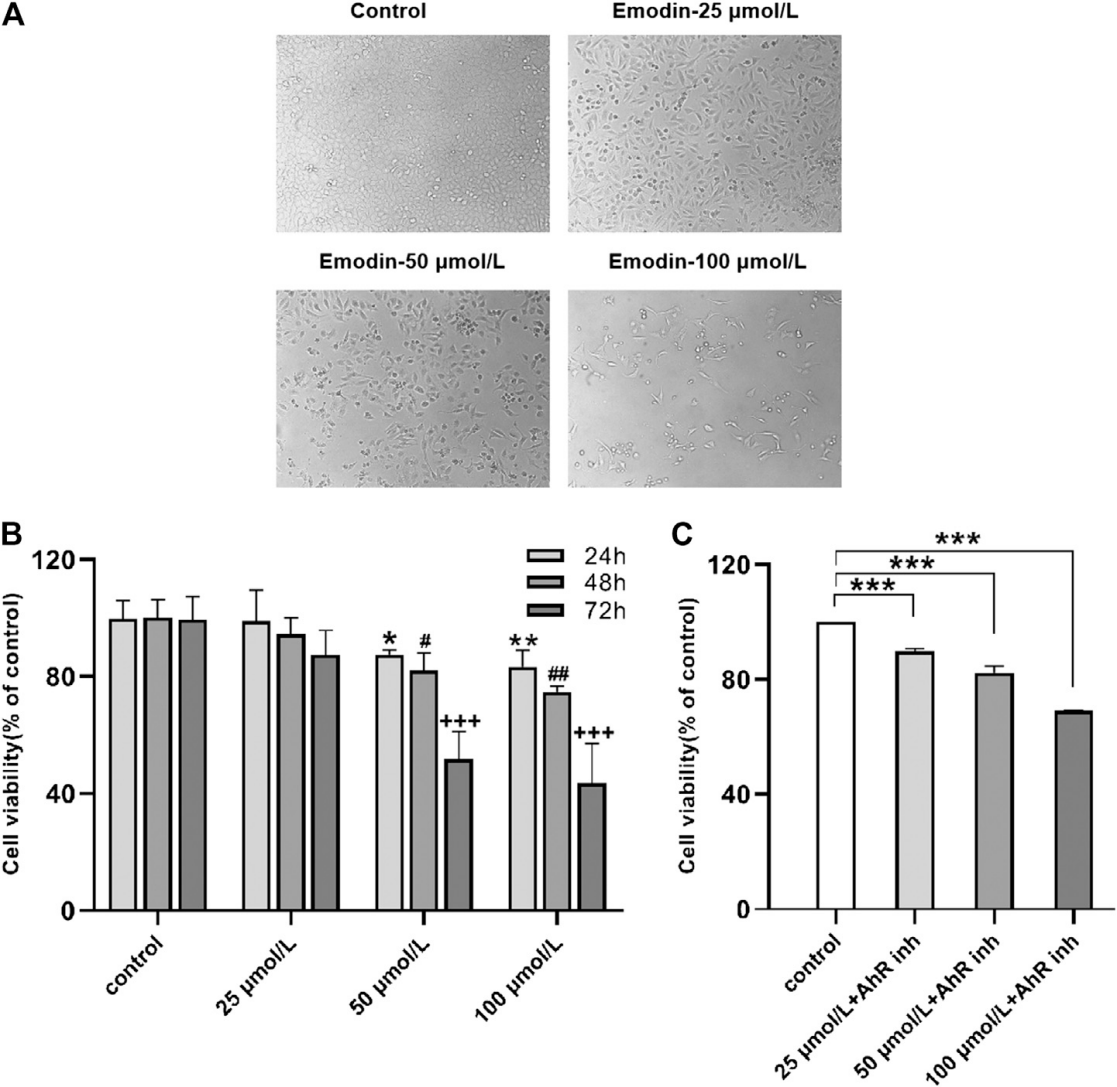
**(A)** Emodin inhibited MCF-7 cell proliferation and growth. Cell morphology under different concentrations of emodin **(B)** MTT assays showed the effects of emodin on MCF-7 cell viability. Values represent mean ± SD, n = 3. *p < 0.05, **p < 0.01, ***p < 0.001 vs. control group (24 h); #*p* < 0.05, ##*p* < 0.01, ###*p* < 0.001 vs. control group (48 h); +*p* < 0.05, ++*p* < 0.01, +++*p* < 0.001 vs. control group (72 h) **(C)** MTT assays showed the effects of emodin containing 10 μmol/L of CH223191 on MCF-7 cell viability. Values represent mean ± SD, n = 3. *p < 0.05, **p < 0.01, ***p < 0.001 vs. control group (72 h).

When viewed with an inverted fluorescence microscope, the MCF-7 cells grew vigorously and appeared mostly oval in shape with intact cell bodies in the normal control group. After treating the MCF-7 cells with increasing concentrations of emodin and longer exposure times, the number of living cells was significantly reduced, the cytoplasm size shrank, and the cells were no longer connected to fragments (Figure 2A), the results proved that emodin can cause cell death.

### Cell Proliferation Detected by MTT Assay

The effects of emodin on the activity of MCF-7 cells were detected by MTT assay. The results indicated that treating MCF-7 cells with different concentrations of emodin resulted in reduced cell proliferation rates. Interestingly, the inhibitory effect of emodin on the proliferation of MCF-7 cells was both time-dependent and concentration-dependent. After treating the cells with emodin, the time of maximum inhibition occurred at 72 h and the concentration of maximum inhibition was 100 μmol/L (Figure 2B). Next, we co-treated the cells with different emodin concentrations and 10 μmol/L of AhR inhibitor for 72 h. The inhibitory effect of emodin on cell proliferation was weakened when the cells were co-treated with the inhibitor (Figure 2C). These experimental results suggested that emodin inhibited the proliferation of breast cancer cells by activating on AhR.

### Flow Cytometric Analysis of Apoptosis

**FIGURE 3:**
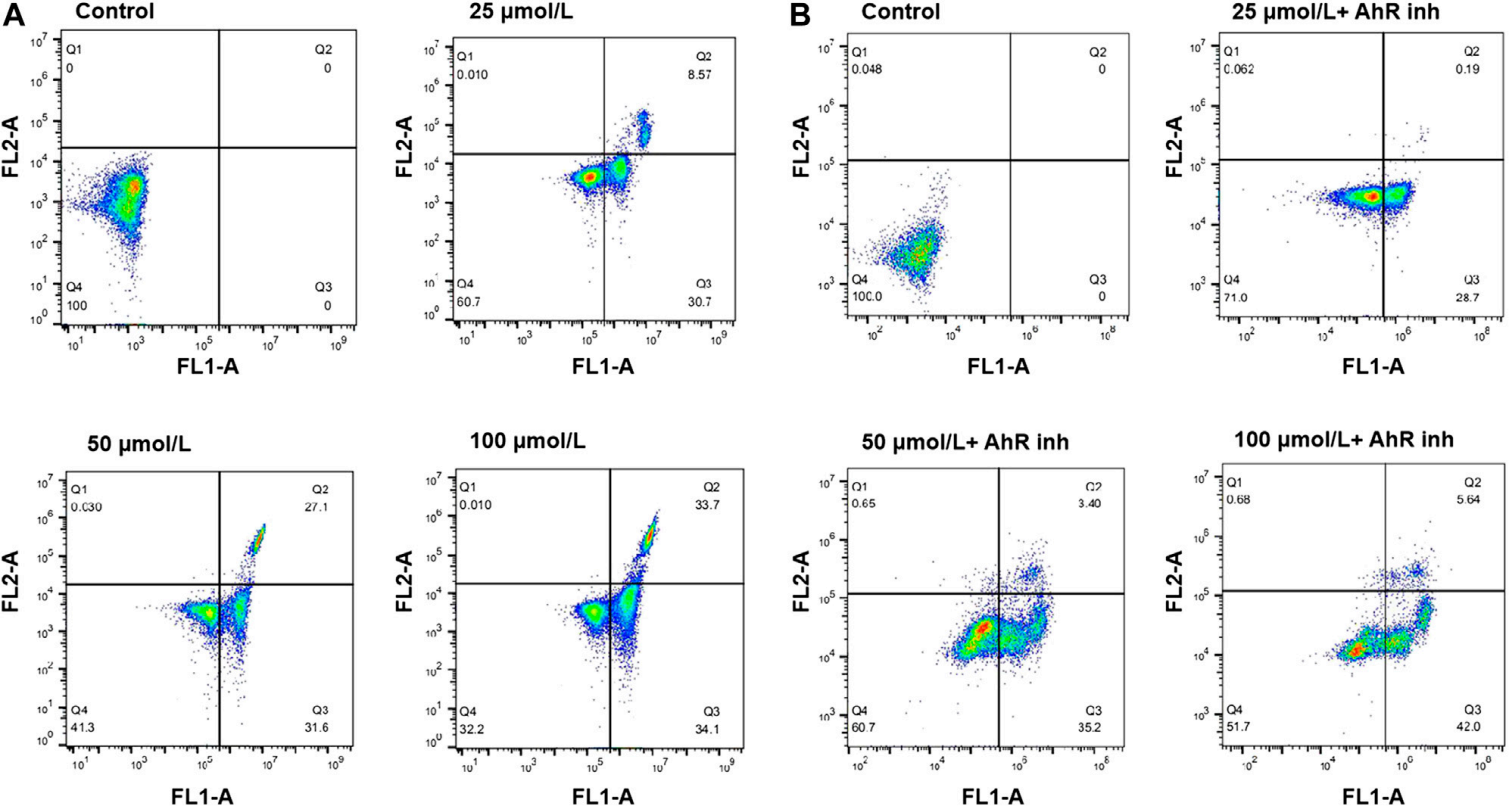
**(A)** Flow cytometry analysis of apoptosis using Annexin V-FITC/PI double staining in MCF-7 cells treated with emodin at the indicated concentrations for 72 h **(B)** Flow cytometry analysis of apoptosis using Annexin V-FITC/PI double staining in MCF-7 cells treated with emodin at the indicated concentrations or emodin with containing 10 μmol/L of CH223191 for 72 h. The percentage of cells is shown in the respective quadrants.

To investigate if the cell death induced by emodin was related to apoptosis, the interaction of MCF-7 cells with emodin was further analyzed by annexin V-FITC/PI staining, and the apoptosis ratios were detected by flow cytometry. After 72 h of incubation with emodin, cells were collected for apoptosis analysis. As shown in Figure 3, after exposure to different concentrations of emodin (25, 50, and 100 μmol/L) in MCF-7 cells, the amount of apoptotic cells (including the early and late apoptosis ratios) increased gradually from 0% relative to the control to 39.27, 58.7, and 67.8%, at the three respective concentrations. When MCF-7 cells were exposed to different concentrations of emodin (25, 50, 100 μmol/L) together with 10 μmol/L of CH223191, the apoptotic cells (including the early and late apoptosis ratios) increased gradually from 0% relative to the control to 28.89, 38.6, and 47.64%, respectively. These results showed that emodin could inhibit the proliferation of breast cancer cells through apoptosis pathway.

### Chemical Structure and Pharmacokinetic Parameters of Emodin

**FIGURE 4:**
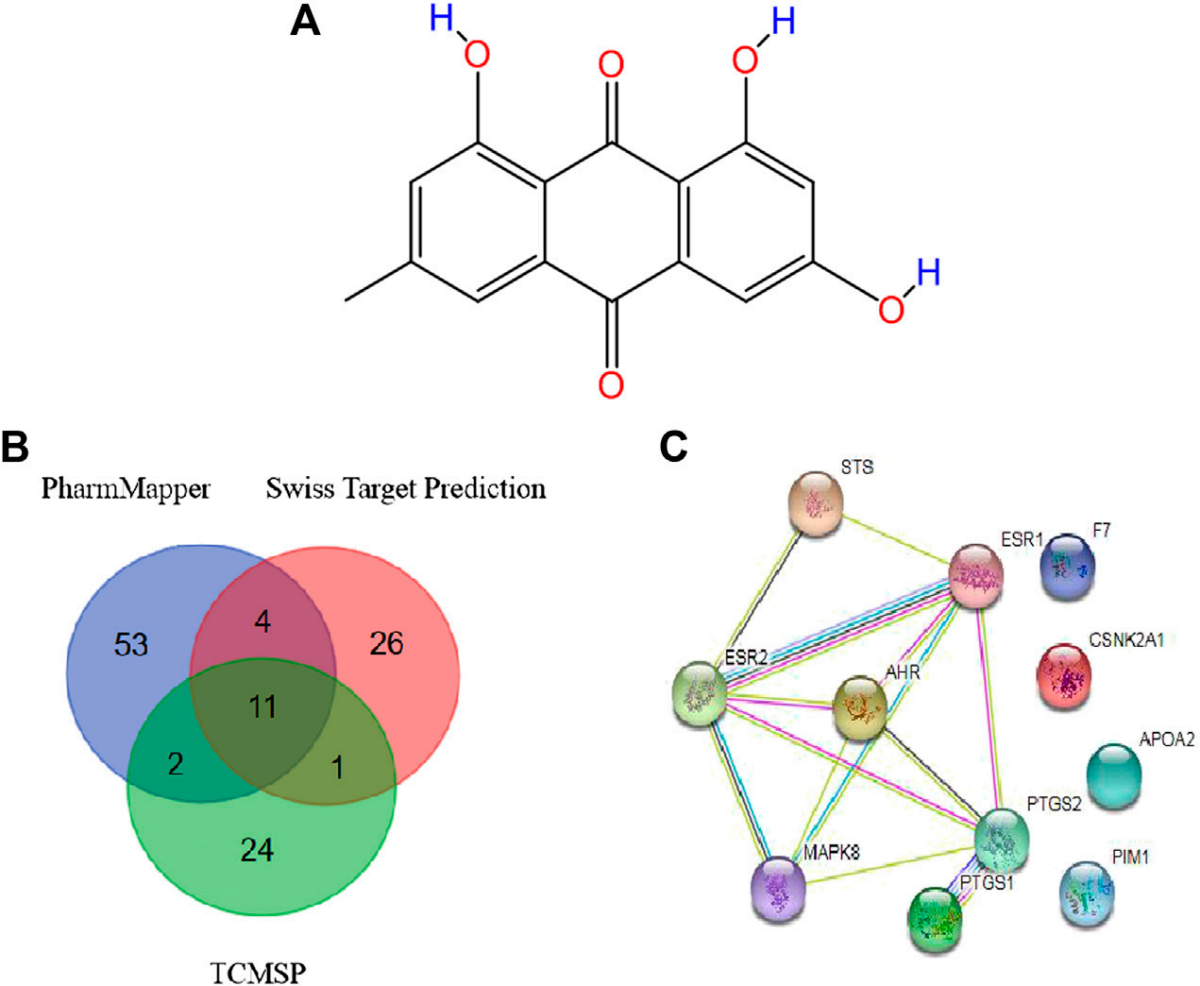
**(A)** Structure of emodin **(B)** Potential protein targets of emodin obtained using the PharmMapper, Swiss Target Prediction, and TCMSP databases **(C)** PPI network of core targets.

The chemical structure information of emodin was obtained from the NCBI PubChem database (Figure 4A), which showed an orange-yellow long needle-like crystal (crystal in acetone was orange, crystal in methanol was yellow). As shown in Table 1, the numerical values of the pharmacokinetic parameters of emodin were acquired by entering the CAS number of emodin into the TCMSP server. The combination of oral bioavailability (OB) screening and drug-likeness (DL) performance evaluation has been widely used to screen properties of compounds (Tian et al., 2015). The OB and DL of emodin were evaluated and showed a high DL value of 0.24, which is higher than the average DL value of 0.18. Therefore, emodin may be a promising drug. Previous studies reported that emodin could act on the digestive and circulatory systems through pharmacological effects including regulation of gastrointestinal function, cardio-cerebrovascular protection, anti-tumor effects, and immune regulation. Therefore, by examining potential targets of emodin, we explored the effects of using this compound on MCF-7 cells and its mechanism of action.

**TABLE 1:** Properties of emodin.

Name	MW	AlogP	Caco-2	OB(%)	DL
Emodin	270.25	2.49	0.22	24.40	0.24

MW: Molecular weight, AlogP: Predicted octanol/water partition coefficient, Caco-2: Predicted apparent Caco-2 cell permeability, OB: oral bioavailability, DL: drug-likeness.

### Target Acquisition

Data were collected by a comprehensive predictive method from three databases, including PharmMapper, Swiss Target Prediction, and TCMSP. Duplicates were removed, a total of 70 potential targets were obtained from the PharmMapper data, and a total of 42 potential targets were obtained from the Swiss Target Prediction data. Meanwhile, we obtained a total of 38 potential targets from the TCMSP data. To improve the specificity of the target protein, we selected 11 interrelated protein targets as candidates for further research by combining the above three databases (Table 2). According to the candidate targets, a Venn diagram was generated (Figure 4B).

**TABLE 2:** Interrelated protein target information.

Rank	UniProtKB	Gene symbol	Gene ID	Protein name
1	P45983	MAPK8	5,599	Mitogen-activated protein kinase 8
2	P08842	STS	412	Steryl-sulfatase
3	P03372	ESR1	2,099	Estrogen receptor alpha
4	Q92731	ESR2	2,100	Estrogen receptor beta
5	P23219	PTGS1	5,742	Prostaglandin G/H synthase 1
6	P35354	PTGS2	5,743	Prostaglandin G/H synthase 2
7	P35869	AhR	196	Aryl hydrocarbon receptor
8	P02652	APOA2	336	Apolipoprotein A-II
9	P11309	PIM1	5,292	Serine/threonine-protein kinase PIM1
10	P08709	F7	2,155	Coagulation factor VII
11	P68400	CSNK2A1	1,457	Casein kinase II subunit alpha

### Construction of a Protein-Protein Interaction (PPI) Network

To develop a better understanding of the association between potential protein targets, the candidate targets were imported into the STRING database to obtain their potential interaction relationships. The interaction network had 11 nodes and 13 edges. However, only seven targets were interconnected among these candidate targets: Mitogen-activated protein kinase 8 (MAPK8), Aryl hydrocarbon receptor (AhR), Estrogen receptor alpha (ESR1), Prostaglandin G/H synthase 2 (PTGS2), Steryl-sulfatase (STS), Prostaglandin G/H synthase 1 (PTGS1), and Estrogen receptor beta (ESR2) (Figure 4C). Therefore, these top seven targets of high-node degrees were considered as the key targets and were the focus of further research.

### GO and KEGG Pathway Enrichment Analyses

**FIGURE 5:**
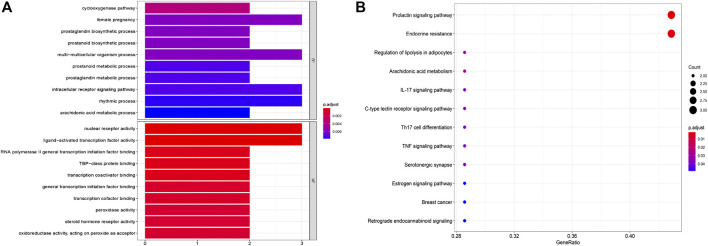
GO and KEGG pathways enrichment analysis of candidate targets. **(A)** The biological processes and molecular function analysis of potential targets. **(B)** The enrichment analysis of the KEGG signaling pathways.

To further investigate the anti-tumor mechanisms of emodin, GO biological function annotation and KEGG signal pathway analyses were carried out on 11 key targets using the DAVID Bioinformatics Resources 6.8 software. GO results showed that the intersection gene set was enriched in 205 biological process pathways and 32 processes related to molecular function (Figure 5A). The 12 KEGG pathways were obtained after running 11 common targets in R language. The results were displayed as a bar graph of KEGG function enrichment, as shown in Figure 5B, and showed that common targets were mainly enriched in the prolactin signaling pathway, endocrine resistance, estrogen signaling pathway, and breast cancer, among other pathways. Through a literature search, we found that the AhR protein plays an important role in the abovementioned diseases. Therefore, we chose AhR as the key target of emodin in breast cancer for more in-depth research.

### Molecular Docking Study

**FIGURE 6:**
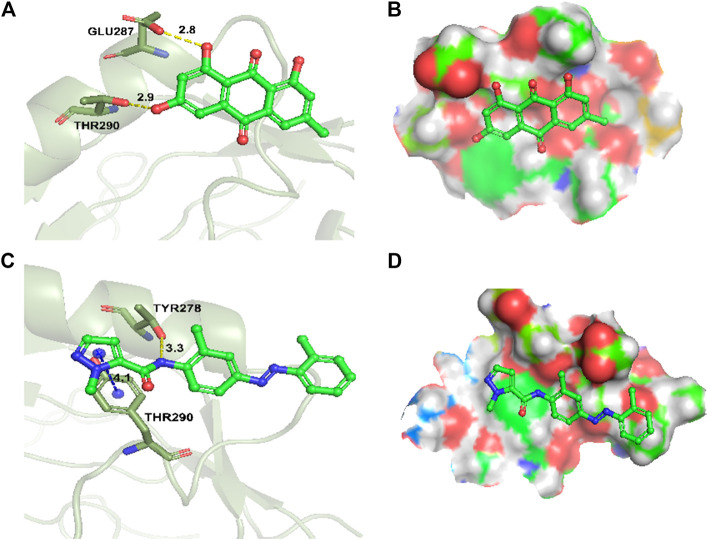
Docking and binding pattern into AhR active site for compounds emodin **(A,B)**, AhR inhibitor CH223191 **(C,D)**. Hydrogen bonds–yellow lines, Pi-cation–blue lines.

To explore the binding mode of emodin and CH223191 with AhR, a molecular docking study was performed using Glide modules of Schrödinger suite 2009 (Schrödinger LLC, New York, United States). The 3D crystal structure of HIF2α (PDB ID: 3F1O) was used for this docking study. Initially, the protein was also docked with CH223191 to perform the verification process, after which emodin was docked using the same protocol. The binding mode of emodin is pictured in Figure 6A,B and that of CH223191 is pictured in Figure 6C,D. Visual inspection of the results indicated that emodin was embedded into the ATP binding pocket of the AhR protein. Moreover, two hydrogen bonds formed within the active binding site of AhR: the AhR Glu 287 residue with the OH-group of emodin and the AhR Thr 290 residue with the OH-group of emodin. The combination of AhR and CH223191 showed that one hydrogen bond formed within the active binding site of AhR: the AhR Thr 290 resided with the N-atom of CH223191. Analysis of the binding mode gave us an explanation for different effects on the AhR activity between the two compounds.

### Western Blot Analysis

**FIGURE 7:**
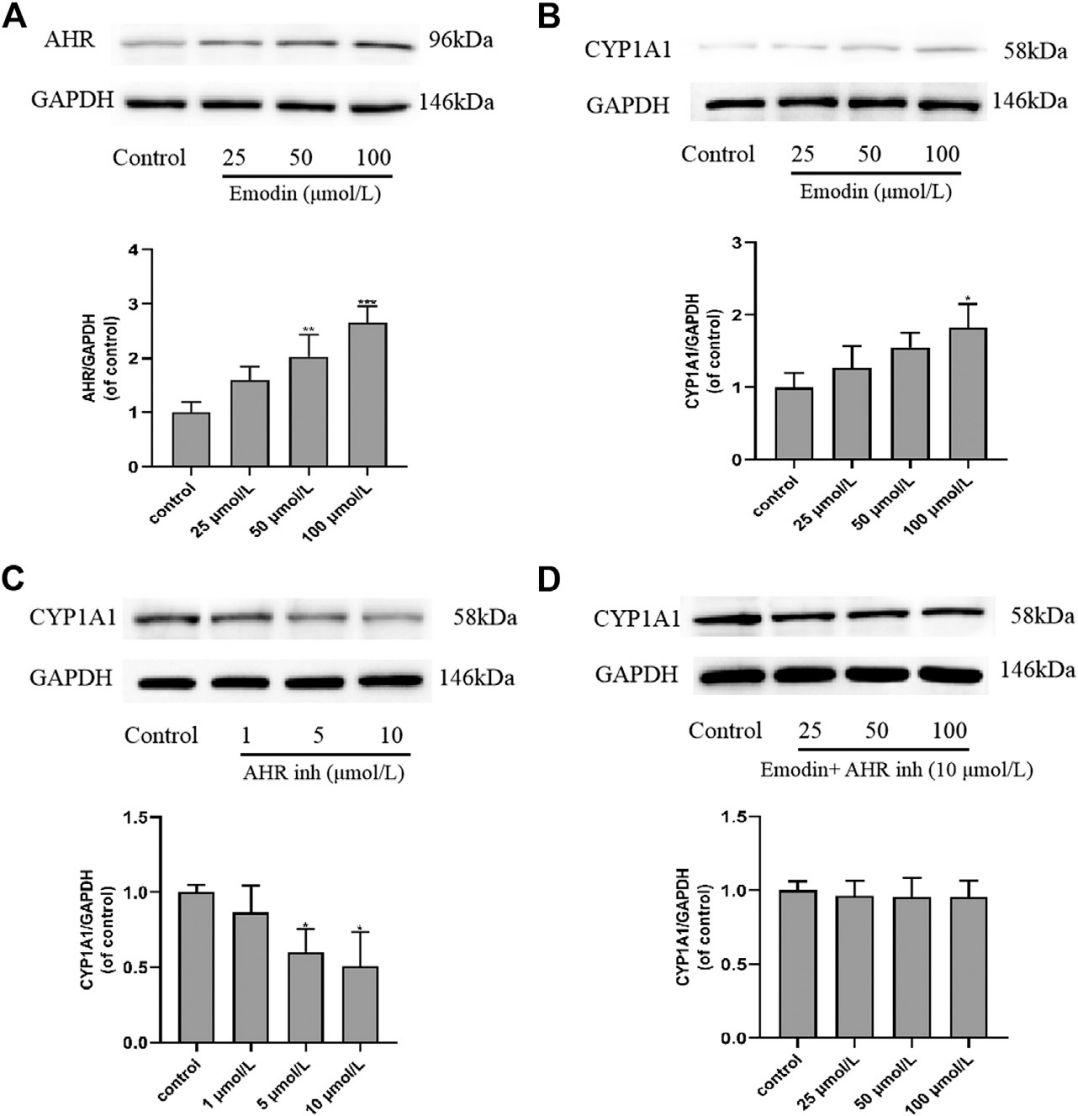
Effects of emodin and AhR inhibitor CH223191 on the protein expression levels of AhR and CYP1A1 in MCF-7 cells (mean ± SD, n = 3) **(A)** In the cells treated with emodin, the expression of AhR protein using GAPDH as a loading control and the graphical representations of the AhR/GAPDH ratio; **(B)** In the cells treated with emodin, the expression of CYP1A1 protein using GAPDH as a loading control and the graphical representations of the CYP1A1/GAPDH ratio; **(C)** In the cells treated with CH223191, the expression of CYP1A1 protein using GAPDH as a loading control and the graphical representations of the CYP1A1/GAPDH ratio; **(D)** In the cells intervened with emodin and 10 μmol/L of CH223191, the expression of CYP1A1 protein using GAPDH as a loading control and the graphical representations of the CYP1A1/GAPDH ratio.

MCF-7 cells were incubated with different concentrations of emodin and CH223191 for 24 h, after which expression levels of AhR and CYP1A1 were further studied by western blot. The results showed that 25 μmol/L of emodin could increase the expression of CYP1A1 compared with the control group. CYP1A1 is the downstream target gene of AhR in MCF-7 cells. With increasing emodin concentration, CYP1A1 protein expression also gradually increased (*p* < 0.05), which was consistent with AhR protein expression (Figure 7A,B). When CH223191 was added and its concentration was increased, CYP1A1 expression decreased (Figure 7C), likely because the expression of CYP1A1 in cells is regulated by AhR. To validate this hypothesis, we co-treated cells with emodin and CH223191. The results suggested that the expression levels of CYP1A1 did not change significantly (Figure 7D). These western blot findings strongly suggested that emodin regulated the expression of AhR and CYP1A1 proteins in MCF-7 cells to inhibit cell proliferation.

## Discussion

This study aimed to investigate the role of AhR in the effects of emodin treatment of MCF-7 breast cancer cells. AhR is a ligand-binding transcription factor that regulates various biological processes (Beischlag et al., 2008; Furness and Whelan, 2009; Marinkovic et al., 2010; Whelan et al., 2010). Studies have found that many drugs used as AhR activators have certain anti-cancer activities. In our study, emodin treatment resulted in concentration-dependent cell death in MCF-7 cells. The MTT results showed that the survival rate of cells decreased in an emodin concentration-dependent manner. When AhR inhibitor CH223191 was added, the survival rate of the cells was improved than cell survival rate of emodin alone (Figure 2). The results of flow cytometry showed that emodin treatment increased the apoptosis rate of MCF-7 cells. However, when CH223191 was added with emodin, the apoptosis rate of the cells decreased. AhR protein expression increased in an emodin concentration-dependent manner. However, when CH223191 was added, AhR expression was significantly reduced (Figure 3). These different experimental results all suggested that emodin acted as an agonist of AhR. When AhR is activated, it can effectively inhibit the growth of cancer cells. This is consistent with the results of previous studies. For example, O'Donnell et al. found that raloxifene was used as an AhR activator to selectively induce apoptosis in triple-negative breast cancer cells (O’Donnell et al., 2014). Huang et al. determined that aloe-emodin inhibited breast cancer cell proliferation through inhibition of the estrogen receptor (Huang et al., 2013). Marconett et al. reported that indole-3-carbinol can be used as an AhR agonist to eliminate estrogen receptors to inhibit breast cancer cell proliferation (Marconett et al., 2010).

Many studies have shown that AhR can regulate the expression of CYP1A1 protein (Li et al., 1998; Zhang et al., 2003; Beischlag et al., 2008). CYP450s are a class of phase I enzymes involved in the metabolism of endogenous and exogenous compounds *in vivo*. They are mainly found in the endoplasmic reticulum of cells and belong to a mixed-function oxidase system (Wei et al., 1996). CYP1A1 is one of the main cytochrome P450 enzymes. Of the different reactions catalyzed by CYP1A1, the hydroxylation of the aromatic ring vacancy position is considered to be a sign of cancer. It can induce cancer-causing mutations by forming highly reactive conversion products (Buterin et al., 2000). CYP1A1 plays an important role in the detoxification of environmental carcinogens and the metabolic activation of dietary compounds with cancer preventive activity. The transcriptional activation of the CYP1A1 gene is mediated by chemical substances that bind to the cytosolic receptor AhR (Hankinson, 1995). In our study, western bolt results showed that emodin activates AhR to increase the expression of CYP1A1 protein. When AhR is specifically inhibited, the expression of CYP1A1 protein decreased. On this basis, emodin and CH223191 were added at the same time, and we found that CYP1A1 expression did not change significantly. These results are consistent with the findings of Wei et al. and Lin et al. (Li et al., 1998; Lin et al., 2003). This suggests that the expression of CYP1A1 protein is indeed regulated by AhR.

Emodin is a flavonoid compound extracted from traditional Chinese medicine rhubarb, polygonum cuspidatum, and aloe (Yang et al., 2014b; Cao et al., 2017). Previous reports have shown that there are other flavonoid compounds that can selectively inhibit breast cancer cells by activating the AhR pathway. The aminoflavonoids studied by Brantley et al. inhibited the growth of breast tumors by activating the AhR target and inhibited α6-integrin (Brantley et al., 2016). Wang et al. showed that *β*-naphthol flavonoids act as AhR agonists and can mediate the cell cycle arrest of estrogen receptor-positive breast cancer through AhR-dependent regulation. They thereby have anti-tumor activity against breast cancer (Wang et al., 2014). It is worth noting that these flavonoids are based on their own structural characteristics, chemical nature, and function as an AhR agonist to play an anti-tumor effects. On this basis, we generated a docking model of emodin and AhR to provide a potential explanation for the anti-tumor effects of this drug as an AhR agonist.

However, this study does have some limitations. For example, we only studied one breast cancer cell line (MCF-7) and did not conduct research and discussion on other breast cancer cell lines. In the MCF-7 cell experiments, we clarified that emodin inhibits cell proliferation and induces apoptosis by activating the AhR/CYP1A1 pathway. On the basis of the research method of network pharmacology, this article can only find the relationship between the target and the disease in the existing databases, but cannot discover new targets. The complex mechanism of action between AhR/CYP1A1 and breast cancer still requires further study.

Overall, emodin may exhibit anti-tumor activity by activating the AhR/CYP1A1 pathway, which lays the foundation for the application of emodin in breast cancer treatment. This research combines network pharmacology methods with molecular docking to screen and determine the targets for the treatment of breast cancer, which provides new ideas for future breast cancer research.

## Data Availability Statement

The original contributions presented in the study are included in the article/Supplementary Material, further inquiries can be directed to the corresponding authors.

## Author Contributions

SM, GH, and NZ conceived of the study. NZ, JW, AS, SH, and YT carried out the experiments and performed the statistical analysis. SM, GH, NZ, and JW performed the coordination and wrote the paper. All authors reviewed and approved the final manuscript.

## Funding

This work was supported by national major science and technology special project for “significant new drugs development” (2019ZX09721001–006–001), the medical and health science and technology innovation project of Chinese Academy of Medical Science (2019-I2M-1-005) and the major natural science research projects in Anhui Universities (KJ2020ZD011).

## Conflict of Interest

The authors declare that the research was conducted in the absence of any commercial or financial relationships that could be construed as a potential conflict of interest.

## Figures and Tables

**TABLE 3:** Molecular docking studies of compounds emodin and AhR inhibitor CH223191 with AhR.

Comp	Glide energy (kcal/mol)	Amino acid residues (within a radius of 4Ǻ from bound ligand)	H-bond formatting residue and bonds
Emodin	−4.064	Tyr316, Leu310, Tyr278, Glu279, Phe280, Tyr281, Hie282, Ala283, Leu284, Ser286, Glu287, Thr290	Thr290 = 2.160, Glu287 = 1.840
AhR inhibitor	−2.740	Asp251, Ser276, Tyr278, Glu279, Tyr291, Hie292, Ala293, Leu294, Asp295, Ser296, Glu297, Asn298, Thr290, Lys291, His293, Gln294	Thr290 = 2.460
